# Interleukin-27: a novel biomarker in predicting bacterial infection among the critically ill

**DOI:** 10.1186/s13054-015-1095-2

**Published:** 2015-10-30

**Authors:** William J. Hanna, Zachary Berrens, Travis Langner, Patrick Lahni, Hector R. Wong

**Affiliations:** Division of Critical Care Medicine, Cincinnati Children’s Hospital Medical Center and Cincinnati Children’s Research Foundation, 3333 Burnet Avenue, Cincinnati, OH 45229 USA; Department of Pediatrics, University of Cincinnati College of Medicine, 3333 Burnet Avenue, Cincinnati, OH 45229 USA

## Abstract

**Introduction:**

A continued need exists for effective diagnostic biomarkers in bacterial sepsis among critically ill patients, despite increasing use of available biomarkers such as procalcitonin (PCT). Interleukin-27 (IL-27) has shown early promise in a recent preliminary study, exhibiting high specificity and positive predictive values for bacterial infection in critically ill children. This validation study was performed to assess the value of IL-27 in predicting bacterial infection among patients admitted to the pediatric intensive care unit and to compare its performance with that of PCT.

**Methods:**

A single-center (n = 702) prospective study was performed comparing both IL-27 and PCT levels between bacterially infected and uninfected cohorts in the pediatric intensive care unit. Infected status was determined by a chart review by an intensivist blinded to biomarker results. Formal performance comparisons included calculations of receiver operating characteristic (ROC) curves for IL-27 and PCT individually in addition to a combination strategy using a decision tree generated by classification and regression tree (CART) methodology. Secondary analysis focusing on subjects with documented bloodstream infections was performed.

**Results:**

The overall infection rate was 27 %. ROC curves for the primary analysis yielded areas under the curve (AUCs) of 0.64 (0.59 to 0.68) for IL-27 and 0.61 (0.56 to 0.65) for PCT. Secondary analysis defining infected status exclusively through positive blood cultures yielded AUCs of 0.75 (0.68 to 0.81) for IL-27 and 0.64 (0.57 to 0.71) for PCT, with a specificity of 95 % (92 % to 97 %) for the prior established IL-27 cut-point value of at least 5.0 ng/ml. Similar AUCs were found for the subset of immunocompromised patients. In a CART-derived analysis taking immunocompromised status into consideration, a combination of IL-27 and PCT yielded an AUC of 0.81 (0.75 to 0.86), statistically improved from either IL-27 or PCT alone.

**Conclusions:**

Despite having a modest predictive value for infection independent of source, IL-27 may serve as a useful biomarker in estimating risk of bacterial infection among critically ill pediatric patients with bloodstream infections. In particular, among immunocompromised subjects, this diagnostic biomarker may be helpful either alone or using a combination strategy with other available biomarkers.

**Electronic supplementary material:**

The online version of this article (doi:10.1186/s13054-015-1095-2) contains supplementary material, which is available to authorized users.

## Introduction

Sepsis is thought to be the leading cause of death in the pediatric population, and mortality was recently estimated to be 1.6 million infants and children per year worldwide. In the US alone, more than 75,000 cases of pediatric severe sepsis are estimated annually with an in-hospital mortality of 14.4 % [1–5]. Although the gold standard for diagnosing sepsis remains positive microbiological cultures, delays from time of acquisition to final result have led many investigators to explore the use of sepsis diagnostic biomarkers as early indicators of infection. In this context, recognizing bacterial forms of sepsis early is critical because it warrants prompt antibiotic therapy, and delays in antibiotic administration in patients with bacterial sepsis have been shown to have negative consequences for outcome. Thus, using bacteria-specific biomarkers for earlier detection, and therefore more timely and appropriate treatment, could prove of great benefit in decreasing both mortality- and morbidity-related to bacterial sepsis [6–9].

Among current biomarkers used in sepsis, procalcitonin (PCT) remains one of the more used, despite its variability in performance depending on patient population and despite a recent meta-analysis in adults showing a lack of reliability in distinguishing infected from uninfected patients in critically ill cohorts [10–12]. Other biomarkers—such as triggering receptor expressed on myeloid cells-1 (TREM-1), soluble urokinase-type plasminogen activator receptor (suPAR), and CD64 [13, 14]—continue to be tested. Recent studies suggest interleukin-27 (IL-27) as another candidate sepsis diagnostic biomarker [15–17]. IL-27 is a heterodimeric cytokine composed of the Epstein-Barr virus-induced gene 3 (EBI3, also known as IL-27B) and the IL27-p28 subunits [18]. IL-27 is produced by antigen-presenting cells upon exposure to microbial-derived molecules and inflammatory stimuli [19–21].

Using a large genome-wide expression database of critically ill children admitted to pediatric intensive care units (PICUs) across the US, 100 class predictor genes, differentially expressed between patients with and without bacterial sepsis, were isolated by using computer-assisted image analysis of gene arrays [15]. Of these 100 predictor genes, EBI3, a subunit of IL-27, was discovered as having the highest predictive strength for bacterial infection. Both IL-27 and PCT concentrations were then measured within a cohort of 231 critically ill children. Findings included a specificity and positive predictive value of more than 90 % for bacterial infection in those with IL-27 levels of at least 5 ng/ml performing significantly better than PCT [15]. Given this, the dearth of other pediatric studies investigating the value of IL-27, for this purpose, and the need for more effective biomarkers in bacterial sepsis, we hypothesized that IL-27 can effectively serve as a diagnostic biomarker among the critically ill pediatric population.

## Methods

The study protocol was approved by the Institutional Review Board of Cincinnati Children’s Hospital Medical Center (CCHMC). Following this, we performed a prospective cohort study of patients admitted to the CCHMC PICU with suspected infection between April of 2013 and December 2014.

### Inclusion criteria

Two criteria were required for study eligibility. The first was admission to the PICU with clinical suspicion for infection. This clinical suspicion was defined by the acquisition of a blood culture at any point during admission by the primary ICU team, performed independently and without interference from the research team. This pragmatic approach captures the exact context in which a diagnostic biomarker would be used by clinicians (i.e., patients with a clinical suspicion of bacterial infection), one that may lead to more generalizable results as compared with a study based on more stringently selected patients. The second was the availability of a residual blood sample within 6 hours of blood culture acquisition, obtained via waiver of informed consent. No exclusion criteria were used.

### Study procedures

Using the CCHMC electronic medical record, the investigating team was notified daily of all blood cultures sent within the prior 24 hours in the ICU. It was then determined which of these patients had a residual, otherwise to be discarded, serum sample within this same time frame in the CCHMC clinical laboratory. These samples were obtained from the lab and used to measure both IL-27 and PCT levels by using the magnetic bead multi-plex platform (EMD Millipore Corporation, Billerica, MA, USA) and Luminex 100/200 System (Luminex Corporation, Austin, TX, USA). The IL-27 assay detects the p28 subunit of IL-27. We did not possess the suitable reagents to measure the related cytokine, IL-35, which consists of the EBI3 and IL12A subunits [15, 22].

### Determination of bacterial infection

Bacterial infection was defined by using both laboratory and clinical data. All final patient classifications were determined by using a majority rule among three intensivists, all blinded to biomarker results. Patients designated as “infected” included all patients with clinically relevant positive bacterial microbiological cultures collected within 48 hours of enrollment. For the primary analysis, these cultures included blood, urine, cerebrospinal, pleural, peritoneal, stool, wound, and endotracheal/tracheal tube cultures. Of note, those patients with strong evidence for bacterial infection in the absence of positive cultures were also included in the “infected” designation. These cases included such findings as radiographic evidence (computed tomography scan, chest x-ray, etc.) or physical exam findings strongly suggesting bacterial infection in the absence of positive cultures. All other subjects were classified as “non-infected”.

### Data collection

In addition to the IL-27 and PCT measurements, relevant demographic data collected included age, gender, reason for admission, type of admission (surgical versus medical), presence of co-morbidities, evidence of pre-existing immune suppression, severity of illness scoring using the Pediatric Risk of Mortality III (PRISM III) score, and both source and etiology of infection. Evidence of immune suppression was determined by a chart review by an intensivist blinded to biomarker results and using data such as use of chronic immunosuppressive medications and evidence of conditions commonly associated with immune-dysregulation.

### Sample size calculation

Using data from the previously published preliminary study [15], we proposed a sample size 700 ICU patients. A main goal of this is to estimate the precision of specificity and sensitivity estimates for IL-27 as a biomarker for bacterial infection. Assuming an expected prevalence of approximately 20 % and a specificity of 92 % as reflected in our preliminary study, 700 patients would result in 95 % confidence interval (CI) for the estimated specificity of ± 2 %. The same sample size will also give a 95 % CI of ± 8 % for the sensitivity of 62 %. Given the above preliminary data, we anticipated data collection to span roughly 20 months.

Because PCT is currently being used clinically as a diagnostic biomarker for bacterial infection, the primary analysis also included a comparison of IL-27 performance with PCT performance, using calculations of respective receiver operating characteristic (ROC) curves. For a comparison of the areas under the curve (AUCs) for IL-27 with that for PCT, assuming areas of about 0.80 and 0.75 as found in our preliminary studies, 700 patients were calculated to provide 90 % power to find the difference, assuming an α of 0.05 and a prevalence of bacterial infection of 20 %.

### Statistical analysis

Using SigmaStat Software (Systat Software Inc., San Jose, CA, USA), all continuous variables were presented as median values and categorical variables as percentages. Statistical tests used to compare study cohorts included Pearson chi-square and Mann-Whitney rank sum tests. Multiple logistic regression was performed to assess independent contributions of PRISM III scores and IL-27 levels on infection status. ROC curve analysis was also performed for both IL-27 and PCT. Test characteristics and their respective 95 % CIs were calculated by using diagnostic test statistics provided by the VassarStats Website for Statistical Computation. In our initial report, the AUC for IL-27 for distinguishing bacterial infection from sterile inflammation was 0.81 (95 % CI 0.75–0.87) [15]. Accordingly, we considered *a priori* that successful validation of IL-27 as a sepsis diagnostic biomarker would be reflected by an AUC falling within this CI. Classification and regression tree (CART) analysis (Salford Predictive Modeler version 6.6; Salford Systems, San Diego, CA, USA) was used in the secondary analysis [23].

## Results

### Demographics

Table 1 shows the demographic and clinical characteristics of the study cohort. In total, 702 patients, having a median age of 5.3 (interquartile range of 1.2–13.5) years, were analyzed. Twenty-seven percent (n = 191) of the patients were determined to be infected: 76 % had positive cultures and 24 % negative cultures. Among those with positive cultures, 46 % were isolated from the blood, 24 % from the respiratory tract (including samples from endotracheal aspirates and bronchoalveolar lavage), and 19 % from the urinary tract. Although PRISM III scores were significantly higher among infected compared with uninfected patients, results of multiple regression analysis using both PRISM III scores and IL-27 levels as independent variables suggest that IL-27 is positively related to infection status independently of severity of illness (Wald’s χ^2^ 5.8, *P* = 0.02).

**Table 1:** Demographic characteristics of the study groups

	Infected	Uninfected	*P* value
Age, years (IQR)	8.2 (1.6–15.1)	4.9 (IQR 1.1–12.4)	0.003
% males	51	58	0.13
% immune-suppressed state	27	30	0.37
% post-operative admission	9	13	0.27
% co-morbidities	77	72	0.27
Median IL-27 concentration, ng/ml (IQR)	1.7 (0.9–3.2)	1.2 (0.8–0.6–2.0)	<0.001
Median PCT concentration, ng/ml (IQR)	7.0 (5.7–9.4)	6.4 (5.3–8.0)	<0.001
Median minutes between culture and IL-27 specimen (IQR)	8.2 (1.6–15.1)	4.9 (1.1–12.4)	0.003
Median PRISM III score (IQR)	6 (2.8–12.0)	3 (0–8)	<0.001

*IQR* interquartile range, *IL-27* interleukin-27, *PCT* procalcitonin, *PRISM III* Pediatric Risk of Mortality III

### Primary analysis

**Fig. 1:**
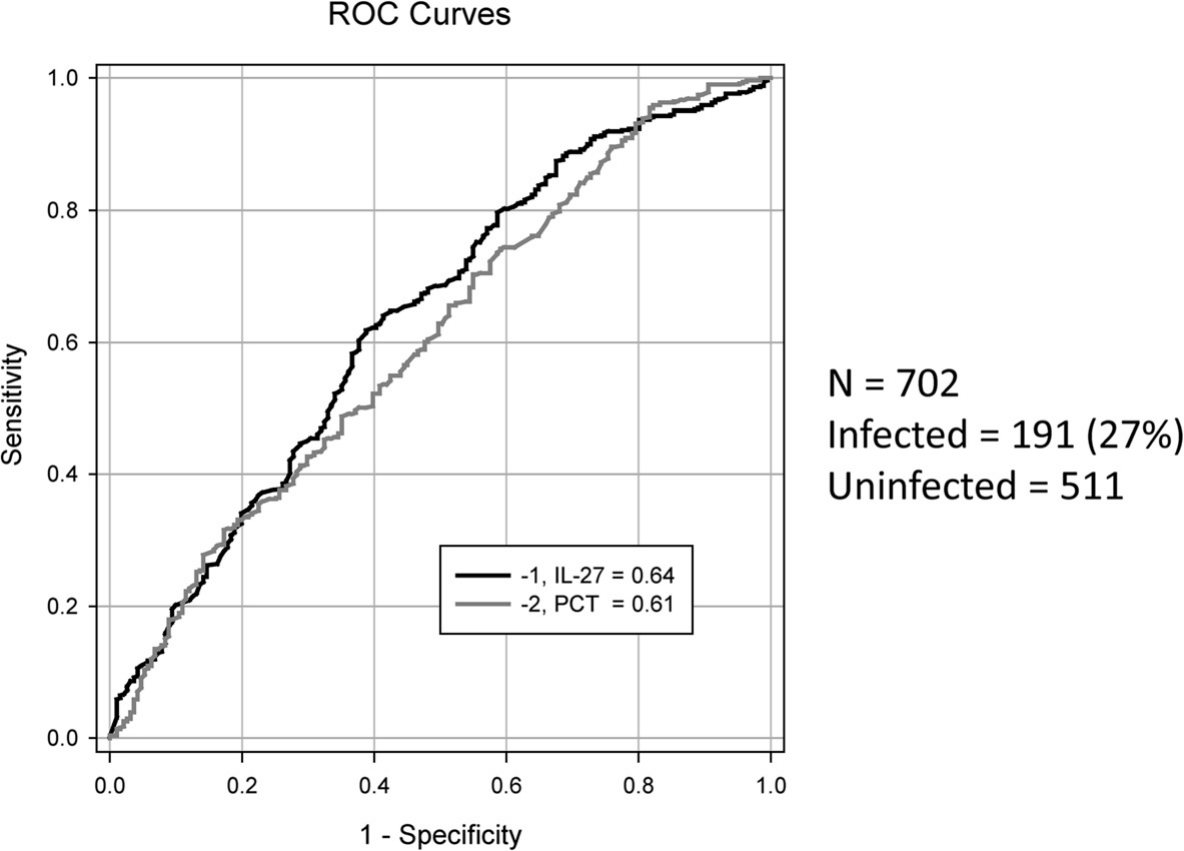
Receiver operating characteristic (ROC) curve for bacterially infected versus uninfected patients in the primary analysis. The procalcitonin (PCT) curve is shown in *grey* and the interleukin-27 (IL-27) curve in *black*

Differences in median values between infected and uninfected groups were significant for both IL-27 (1.7 versus 1.2 ng/ml, *P* <0.001) and PCT (7.0 versus 6.4 ng/ml, *P* < 0.001). ROC curves generated resulted in AUCs of 0.64 (0.59, 0.68, *P* < 0.001) for IL-27 and 0.61 (0.56, 0.65, *P* < 0.001) for PCT. The area difference between the AUCs was not significant. See Fig. 1 for the ROC curve and Table 2 for the associated test characteristics.

**Table 2:** Test characteristics for predicting bacterial infection

IL-27, ≥ng/ml	Sensitivity	Specificity	NPV	PPV
2.0	43 % (36–51)	76 % (72–80)	71 % (67–74)	29 %(26–33)
3.0	27 % (21–34)	91 % (88–93)	77 % (73–80)	52 % (42–63)
4.0	20 % (15–26)	94 % (91–96)	76 % (72–79)	54 % (42–66)
5.0	12 % (8–17)	95 % (93–97)	74 % (71–77)	47 % (32–62)
6.0	8 % (5–13)	96 % (94–98)	74 % (70–77)	46 % (29–63)

*IL-27* interleukin-17, *NPV* negative predictive value, *PPV* positive predictive value

### Secondary analysis

**Fig. 2:**
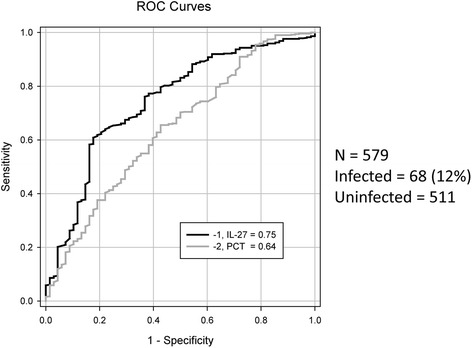
Receiver operating characteristic (ROC) curve for bacterially infected versus uninfected patients in the secondary analysis. The definition of infected includes those patients with blood culture-positive bacterial infections. The procalcitonin (PCT) curve is shown in *grey* and the interleukin-27 (IL-27) curve in *black*

Given that the infected patients in the prior pediatric study primarily included patients with positive blood cultures, a secondary analysis was performed by using a definition of infection that included only patients with clinically relevant positive blood cultures. Five hundred seventy-nine patients were included, 68 (12 %) of whom were classified as infected exclusively on the basis of blood cultures. ROC curves yielded AUCs of 0.75 (0.68–0.81) for IL-27 and 0.64 (0.57–0.71) for PCT, with an area difference of 0.10 (*P* = 0.02) (Fig. 2). Using this definition of infection and the prior cut-point of 5 ng/ml for IL-27, test characteristics included a specificity of 95 % (93–97) and a positive predictive value of 47 % (32–62) (Additional file 1). Further analysis focused on the subclass of patients classified as immune-suppressed (n = 182) revealed similar AUCs of 0.75 (0.64–0.85) and 0.67 (0.56–0.78) for IL-27 and PCT, respectively.

**Fig. 3:**
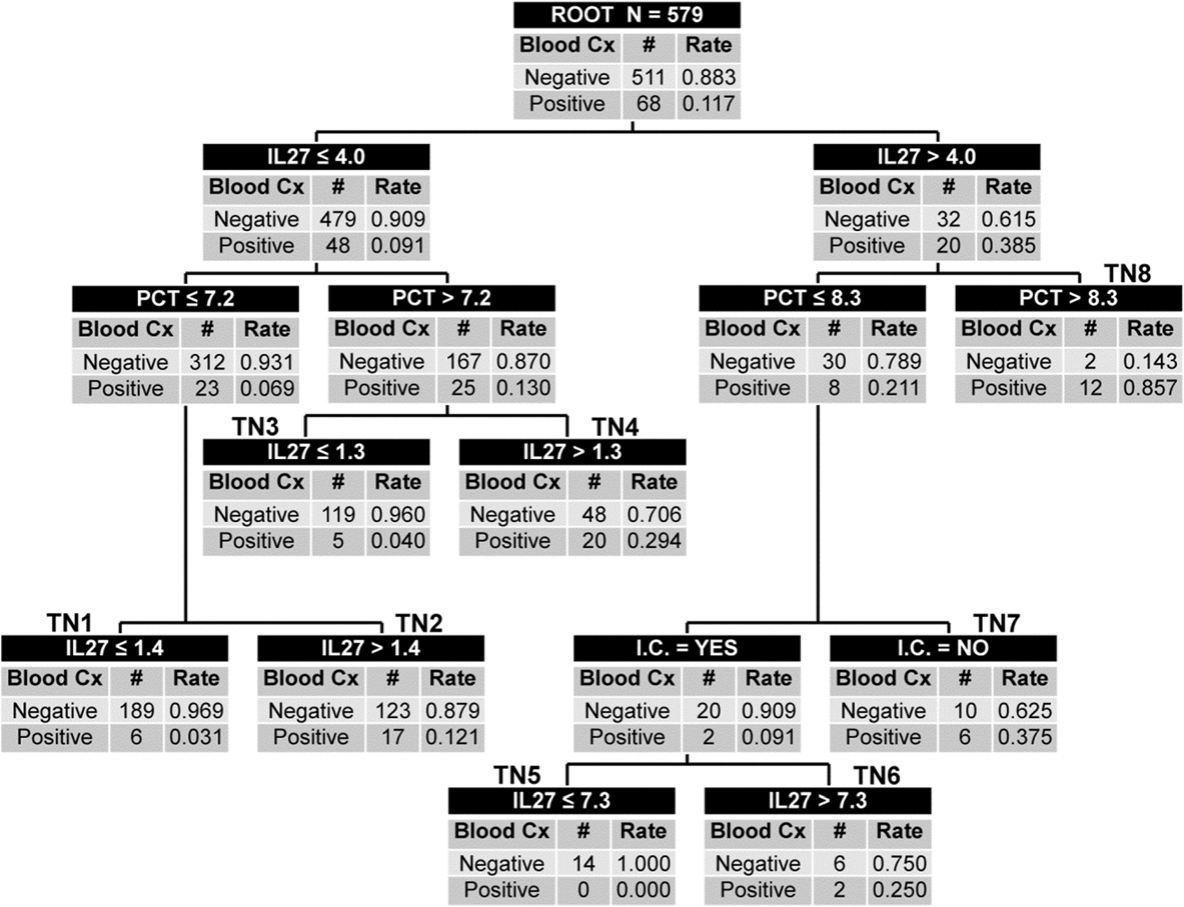
The classification tree includes interleukin-27 (IL-27), procalcitonin (PCT), and immunocompromised status (I.C.) as predictor variables. The biomarker concentrations are presented in nanograms per milliliter. The root node provides the total number of subjects, and the number of subjects with negative and positive blood cultures, with the respective rates. Each daughter node provides the respective decision rule criterion and the number of subjects with negative and positive blood cultures, with the respective rates. Terminal nodes (TN) TN1, TN3, and TN5 are considered low-risk terminal nodes (≤12 % risk of positive culture). TN2, TN4, TN6, TN7 and TN8 are higher-risk terminal nodes (≥12 % risk of positive) compared with the root node

A subsequent CART analysis was generated by using this same modified definition which included IL-27 levels, PCT levels, and immunocompromised status as predictor variables (Fig. 3). Starting with the “root” node that included all patients (n = 579) and dividing into two subsequent “daughter” nodes, eight terminal nodes were ultimately generated, each with specific cutoff points for IL-27 and PCT. Terminal nodes 1, 3, and 5 were designated as low-risk nodes with 3 %, 4 %, and 0 % of patients being infected, respectively. Terminal nodes 2, 4, 6, 7, and 8 were designated as higher-risk nodes compared with the “root” node, with rates of 12 %, 29 %, 25 %, 38 %, and 86 %, respectively. Using these designations, the CART algorithm was then tested, yielding an AUC of 0.81 (0.75, 0.86). This was statistically improved from both the individual IL-27 (0.81 versus 0.74, *P* < 0.01) and PCT (0.81 versus 0.64, *P* <0.01) AUCs (Additional file 2). Test characteristics (95 % CI) for the tree yielded a sensitivity of 84 % (72–91), specificity of 63 % (59–67), negative predictive value of 97 % (94–98), positive predictive value of 23 % (18–29), positive likelihood ratio of 2.3 (1.9–2.6), and negative likelihood ratio of 0.3 (0.2–0.4).

## Discussion

We prospectively tested the diagnostic utility of IL-27 as a sepsis diagnostic biomarker in a heterogeneous cohort of critically ill children. In the primary analysis, both IL-27 and PCT demonstrated poor reliability for estimating bacterial infection risk, as determined by *a priori* criteria based on the previously published ROC curve [15]. A trend to improvement was noted, however, when a secondary analysis was performed by using a modified definition of infection that included only those patients found to have positive blood cultures, and IL-27 was found to have a significantly better predictive value when compared with PCT. With this definition, an IL-27 cutoff value of at least 5 ng/ml was also noted to have a similar diagnostic value as a “rule in” test to the original study, exhibiting a specificity of 95 % [15]. The lower positive predictive value may be explained by a lower prevalence of positive cases, as the prior study exhibited a prevalence of positive cases nearly five times (56 %) the current cohort [15]. Lastly, a CART-generated algorithm that included IL-27, PCT, and immune-compromised status led to a statistically significant trend toward improvement in predictive value.

Our results suggest that IL-27 has a greater predictive value in patients with positive bloodstream infections compared with infections from other body compartments. These results are consistent with the original pediatric study in which the majority of positive cases involved patients with positive blood cultures [15]. Moreover, two recent adult studies have shown improved predictive values of IL-27 when excluding pulmonary sources of infection from the “infected” definition [16, 17]. The biological basis of these findings may be related to increased immune upregulation associated with systemic infection but is unclear and warrants further investigation. Alternatively, given that recent population-based studies indicate a bacterial etiology in only roughly 8 % of children with clinically and radiographically diagnosed pneumonia, concerns of misclassification bias may be more heightened among such populations as these [24]. We have attempted to minimize this concern by using three independent intensivists in the classification process. Nevertheless, by classifying only patients with clinically relevant culture-positive bloodstream infections as “infected” and using those with no concerns for infection as a comparator, this bias may have been further minimized.

Although conclusions are limited by a relatively small number of infected patients, the predictive value of IL-27 among the subset of immunocompromised patients is an interesting finding. That immunocompromised status among other demographic variables was included in the decision tree also suggests IL-27 may yet play a significant diagnostic role in this population. Of note, more than 80 % of those patients designated as immune-compromised were being administered immunosuppressive medications and roughly 50 % had a diagnosis requiring bone marrow or solid organ transplantation. Although IL-27, produced by antigen-presenting cells upon stimulation by microbial products [18, 21], might be thought of as less predictive among populations in which such cells are compromised, such a perspective likely greatly simplifies the picture. Both the complex pleotropic nature of IL-27 and the vast variety of immunopathology combined under the heading of “immune compromised” warrant further elucidation.

Notable limitations to our study include lack of data regarding temporal production of IL-27. By selecting blood samples for analysis no later than 6 hours following blood culture acquisition, we have attempted to minimize this. Median times from blood culture to biomarker specimen acquisition were less than 10 minutes for both infected and uninfected cohorts (Table 1). In addition, the study was not adequately powered to conclusively comment on the predictive trend among immunocompromised patients. Further studies may be warranted in this population. Lastly, among the potential confounding variables, severity-of-illness scores were found to be significantly higher in the infected cohort. However, this was accounted for by regression analysis that established IL-27 levels as an independent predictor of infected status.

## Conclusions

Our results suggest that IL-27 may serve as a useful biomarker in estimating risk of bacterial infection among critically ill pediatric patients with bloodstream infections. In particular, among those classified as immune-compromised, this diagnostic biomarker may be helpful either alone or using a combination strategy with other available biomarkers, although further research is warranted.

## Key messages

- Despite having modest predictive value for all bacterial infections, interleukin-27 may serve as a useful diagnostic biomarker among critically ill children with bloodstream infections.
- Interleukin-27 outperformed procalcitonin in the subgroup of patients with bloodstream infections.
- A combination strategy including both biomarkers may aid in the early diagnosis of bacterial infection among immunocompromised patients.

## Additional files

Additional file 1:
**Test characteristics for predicting bacterial blood culture-positive bacterial infection.** Test characteristics, including sensitivity, specificity, and positive and negative predictive values of the subset of patients defined as infection through the presence of positive bacterial blood cultures. (DOCX 12 kb) Additional file 2:
**ROC comparison of CART model with IL-27 and PCT alone.** ROC curve for blood culture-positive infected versus uninfected patients, including results from the CART analysis. The PCT curve is shown in *black*, IL-27 curve in *grey*, and the CART ROC curve in *green. CART* classification and regression tree, *IL-27* interleukin-27, *PCT* procalcitonin, *ROC* receiver operating characteristic. (TIFF 1707 kb)

## Abbreviations

AUC: Area under the curve
CART: Classification and regression tree
CCHMC: Cincinnati Children’s Hospital Medical Center
CI: Confidence interval
EBI3: Epstein-Barr virus-induced gene 3
ICU: Intensive care unit
IL-27: Interleukin-27
PCT: Procalcitonin
PICU: Pediatric intensive care unit
PRISM III: Pediatric Risk of Mortality III
ROC: Receiver operating characteristic

## References

[1] Black RE, Cousens S, Johnson HL, Lawn JE, Rudan I, Bassani DG (2010). Global, regional, and national causes of child mortality in 2008: a systematic analysis. Lancet.

[2] Hartman ME, Linde-Zwirble WT, Angus DC, Watson RS (2013). Trends in the epidemiology of pediatric severe sepsis. Pediatr Crit Care Med.

[3] Watson RS, Carcillo JA (2005). Scope and epidemiology of pediatric sepsis. Pediatr Crit Care Med.

[4] Watson RS, Carcillo JA, Linde-Zwirble WT, Clermont G, Lidicker J, Angus DC (2003). The epidemiology of severe sepsis in children in the United States. Am J Respir Crit Care Med.

[5] Weiss SL, Fitzgerald JC, Pappachan J, Wheeler D, Jaramillo-Bustamante JC, Salloo A (2015). Global epidemiology of pediatric severe sepsis: the sepsis prevalence, outcomes, and therapies study. Am J Respir Crit Care Med.

[6] Kumar A, Haery C, Paladugu B, Symeoneides S, Taiberg L, Osman J (2006). The duration of hypotension before the initiation of antibiotic treatment is a critical determinant of survival in a murine model of Escherichia coli septic shock: association with serum lactate and inflammatory cytokine levels. J Infect Dis.

[7] Kumar A, Roberts D, Wood KE, Light B, Parrillo JE, Sharma S (2006). Duration of hypotension before initiation of effective antimicrobial therapy is the critical determinant of survival in human septic shock. Crit Care Med.

[8] Weiss SL, Fitzgerald JC, Balamuth F, Alpern ER, Lavelle J, Chilutti M (2014). Delayed antimicrobial therapy increases mortality and organ dysfunction duration in pediatric sepsis. Crit Care Med.

[9] Puskarich MA, Trzeciak S, Shapiro NI, Arnold RC, Horton JM, Studnek JR (2011). Association between timing of antibiotic administration and mortality from septic shock in patients treated with a quantitative resuscitation protocol. Crit Care Med.

[10] Arkader R, Troster EJ, Lopes MR, Junior RR, Carcillo JA, Leone C (2006). Procalcitonin does discriminate between sepsis and systemic inflammatory response syndrome. Arch Dis Child.

[11] Tang BM, Eslick GD, Craig JC, McLean AS (2007). Accuracy of procalcitonin for sepsis diagnosis in critically ill patients: systematic review and meta-analysis. Lancet Infect Dis.

[12] Gibot S, Bene MC, Noel R, Massin F, Guy J, Cravoisy A (2012). Combination biomarkers to diagnose sepsis in the critically ill patient. Am J Respir Crit Care Med.

[13] Sandquist M, Wong HR (2014). Biomarkers of sepsis and their potential value in diagnosis, prognosis and treatment. Expert Rev Clin Immunol.

[14] Standage SW, Wong HR (2011). Biomarkers for pediatric sepsis and septic shock. Expert Rev Anti Infect Ther.

[15] Wong HR, Cvijanovich NZ, Hall M, Allen GL, Thomas NJ, Freishtat RJ (2012). Interleukin-27 is a novel candidate diagnostic biomarker for bacterial infection in critically ill children. Crit Care.

[16] Wong HR, Lindsell CJ, Lahni P, Hart KW, Gibot S (2013). Interleukin 27 as a sepsis diagnostic biomarker in critically ill adults. Shock.

[17] Wong HR, Liu KD, Kangelaris KN, Lahni P, Calfee CS (2014). Performance of interleukin-27 as a sepsis diagnostic biomarker in critically ill adults. J Crit Care.

[18] Pflanz S, Timans JC, Cheung J, Rosales R, Kanzler H, Gilbert J (2002). IL-27, a heterodimeric cytokine composed of EBI3 and p28 protein, induces proliferation of naive CD4+ T cells. Immunity.

[19] Villarino AV, Larkin J, Saris CJ, Caton AJ, Lucas S, Wong T (2005). Positive and negative regulation of the IL-27 receptor during lymphoid cell activation. J Immunol.

[20] Wirtz S, Tubbe I, Galle PR, Schild HJ, Birkenbach M, Blumberg RS (2006). Protection from lethal septic peritonitis by neutralizing the biological function of interleukin 27. J Exp Med.

[21] Wojno ED, Hunter CA (2012). New directions in the basic and translational biology of interleukin-27. Trends Immunol.

[22] Sun T, Zhang D, Yang Y, Zhang X, Lv C, Fu R (2015). Interleukin 35 may contribute to the loss of immunological self-tolerance in patients with primary immune thrombocytopenia. Br J Haematol.

[23] Che D, Liu Q, Rasheed K, Tao X (2011). Decision tree and ensemble learning algorithms with their applications in bioinformatics. Adv Exp Med Biol.

[24] Jain S, Williams DJ, Arnold SR, Ampofo K, Bramley AM, Reed C (2015). Community-acquired pneumonia requiring hospitalization among U.S. children. N Engl J Med.

