# Supplementary material for: Interleukin-27: a novel biomarker in predicting bacterial infection among the critically ill
Source: Crit Care. 2015 Oct 30;19:378. doi: 10.1186/s13054-015-1095-2 (PMC4627377; doi:10.1186/s13054-015-1095-2)
Supplement: Additional file 1: — Test characteristics for predicting bacterial blood culture-positive bacterial infection. Test characteristics, including sensitivity, specificity, and positive and negative predictive values of the subset of patients defined as infection through the presence of positive bacterial blood cultures. (DOCX 12 kb) [file 13054_2015_1095_MOESM1_ESM.docx]

| **Additional File 1. Test Characteristics for Predicting Blood Culture + Bacterial Infection** | | | | |
| --- | --- | --- | --- | --- |
| **IL-27 (≥ng/ml)** | **Sensitivity** | **Specificity** | **NPV** | **PPV** |
| **2.0** | 72% (55-85) | 71% (63-81) | 94% (86-99)) | 14% (10-46) |
| **3.0** | 51% (42-73) | 89% (82-98) | 93% (84-96) | 44% (23-62) |
| **4.0** | 37% (27-67) | 93% (89-97) | 91% (84-94) | 52% (32-68) |
| **5.0** | 26% (12-48) | 95% (90-98) | 90% (80-94) | 47% (39-66) |
| **6.0** | 21% (9-44) | 96% (93-99) | 89% (79-93) | 50% (40-67) |
