# Supplementary figures and images for: Interleukin-27: a novel biomarker in predicting bacterial infection among the critically ill
Source: Crit Care. 2015 Oct 30;19:378. doi: 10.1186/s13054-015-1095-2 (PMC4627377; doi:10.1186/s13054-015-1095-2)

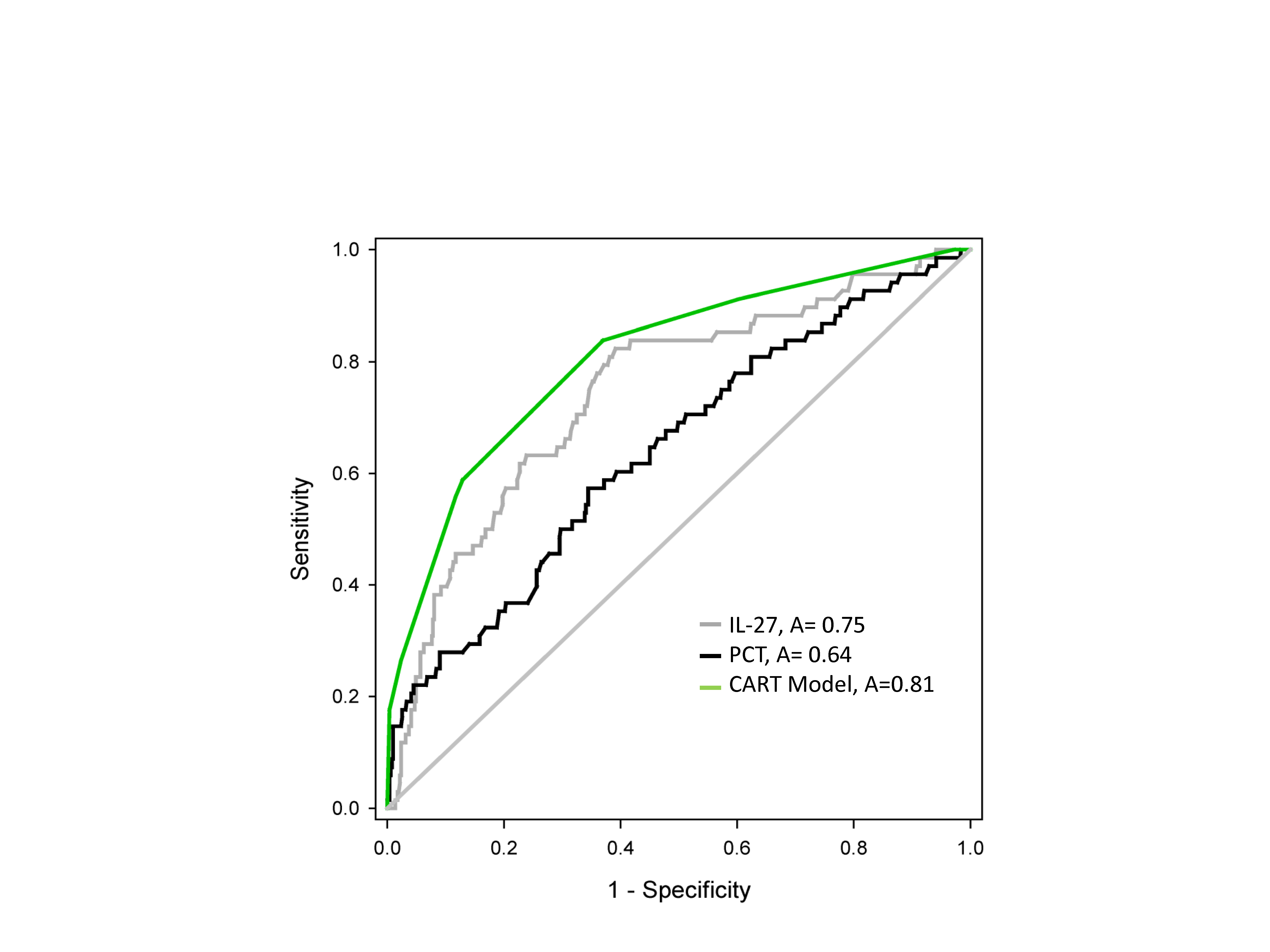

Supplement: Additional file 2: — ROC comparison of CART model with IL-27 and PCT alone. ROC curve for blood culture-positive infected versus uninfected patients, including results from the CART analysis. The PCT curve is shown in black, IL-27 curve in grey, and the CART ROC curve in green. CART classification and regression tree, IL-27 interleukin-27, PCT procalcitonin, ROC receiver operating characteristic. (TIFF 1707 kb) [file 13054_2015_1095_MOESM2_ESM.tiff]
